# An optimized sliding rail-assisted micrometer system for sensing volume measurement of open-ended coaxial probes in breast cancer dielectric property analysis

**DOI:** 10.3389/fbioe.2025.1575142

**Published:** 2025-08-29

**Authors:** Shuai Chen, Zhongliang Lu, Qiang Huang, Gaowei Zhao, Zheng Sun, Jing Zhou, Yupeng Liao

**Affiliations:** ^1^ School of Medical Information Engineering, Gannan Medical University, Ganzhou, China; ^2^ School of Information Engineering, Jiangxi University of Science and Technology, Ganzhou, China; ^3^ Equipment Department, The First Affiliated Hospital of Guangzhou Medical University, Guangzhou, China; ^4^ Department of Pathology, Ganzhou People’s Hospital, Ganzhou, China

**Keywords:** open-ended coaxial probe (OECP) method, biological tissue dielectric properties, sensing depth, sensing radius, heterogeneous tissue

## Abstract

**Objective:**

The open-ended coaxial probe (OECP) method has demonstrated promising potential in biological tissue measurements. However, it still faces challenges such as significant measurement errors and poor repeatability. Research indicates that a substantial portion of these errors originates from tissue heterogeneity. To mitigate errors associated with tissue heterogeneity and accurately interpret the relationship between the dielectric properties and histology of heterogeneous tissue samples, detailed knowledge of the probe’s effective sensing volume is essential.

**Methods:**

In this study, the effective sensing volumes of two commonly used small-aperture probes (with diameters of 2.20 mm and 3.58 mm) were measured. The vertical sensing volume is represented by the sensing depth, while the horizontal sensing volume is characterized by the sensing radius. A measurement model for the sensing volume of the OECP method was established using a heterogeneous dielectric property layered model combined with an optimized sliding rail-assisted micrometer system. Dielectric property bilayer models were constructed using materials with distinct dielectric parameters (Teflon, ethanol, methanol, deionized water) and biological tissue simulants (dimethyl sulfoxide, salt-sugar mixed solution). To validate the sensing volume derived from the aforementioned bilayer model, we conducted experimental measurements on porcine tissue and human breast tissue, both of which exhibit well-defined layered structures. In this experiment, the geometric center of a Teflon cube was designated as the origin for probe movement.

**Results:**

The measured sensing depth ranges were 0.44 to 0.62 mm for a 2.20 mm diameter probe and 0.75 to 0.98 mm for a 3.58 mm diameter probe. While the corresponding sensing radius ranges of 0.36 to 0.63 mm for the 2.20 mm diameter probe and 0.71 to 0.99 mm for the 3.58 mm diameter probe.

**Conclusion:**

The results indicate that both the sensing depth and radius of the probe increase significantly with larger coaxial probe aperture sizes. Furthermore, a smaller aperture reduces the influence of tissue heterogeneity on measurements, while the effective sensing volume remains consistent across frequencies.

## 1 Introduction

Electromagnetic diagnostic technologies (e.g., microwave imaging systems and radiofrequency ablation therapy), which offer advantages such as low cost and minimally invasiveness, have been a popular research direction in biomedical technology (Ahmed et al., 2012; Avery et al., 2017; Deas Yero et al., 2018; Leijsen et al., 2019). An accurate understanding of the dielectric properties of diseased and healthy tissues is an important basis for the development of electromagnetic diagnostic techniques. The dielectric properties of biological tissues mainly include conductivity and relative permittivity, which are the inherent physical properties of biological tissues exhibited under electromagnetic field exposure. Cancer changes the composition and permeability of the cell membrane, potassium, magnesium and calcium move out of the cell while sodium and water accumulate inside the cell, resulting in tumor tissues showing higher dielectric property values (Cone, 1970; Schepps and Foster, 1980). There are several methods for measuring the dielectric property values of biological tissues, including transmission lines, cavities, quadrupole (or multipole) probes and open coaxial probe (OECP) techniques (Wang and Aslani, 2019; Alahnomi et al., 2021; Šarolić and Matković, 2022; Murat et al., 2024; Toro et al., 2024). Among these methods, the open-ended coaxial probe method is the most commonly used due to its ease of construction, wide measurement frequency range, and fast measurement speeds, it has been extensively studied (Cheng and Fu, 2018; La Gioia et al., 2018; Yu et al., 2020; Sasaki et al., 2022).

Although promising results in biological tissue measurements have been demonstrated, the OECP technique suffers from the disadvantages of large measurement errors and poor measurement reproducibility. Studies have shown that most of the larger error margins come from tissue heterogeneity (Gioia et al., 2018). Heterogeneity refers to the organization consisting of objects with two or more layers. Typically, the open-ended coaxial probe method assumes that the sample is homogeneous and the measurement results in the average dielectric properties of the tissue of interest, and the probe should be selected so that the sensing volume is limited to the tissue sample of interest and not to other tissues, in order to reduce the measurement uncertainty due to tissue heterogeneity. To minimize errors related to tissue heterogeneity and to precisely interpret the correlation between the dielectric properties and histological features of heterogeneous tissue samples, detailed knowledge of the probe’s effective sensing volume is essential. Typically, the sensing volume is described by the depth of sensing and the radius of sensing (Meaney et al., 2014; Porter and O'Halloran, 2017). The sensing depth and radius of coaxial probes are determined based on the expected threshold deviation of dielectric constant from a uniform background, measured axially and radially from the probe tip, respectively, under conditions of tissue heterogeneity.

A number of studies on the depth of sensing and radius of sensing of open coaxial probes with different aperture sizes have been reported recently. In the literature (Hagl et al., 2003), Hagl compared two probes with different aperture sizes (2.20 mm and 3.58 mm diameter) to accurately characterize the dielectric properties of breast tissue. The study used ethanol, methanol and deionized water as test materials with the aim of determining the depth of sensing of the two probes. The experimental results demonstrated that for accurate measurements, the minimum sample dimensions should be 3.0 mm in thickness and 1.1 cm in lateral size when using a 3.58 mm diameter probe. For a smaller 2.20 mm diameter probe, the sample thickness and width should be at least 1.5 mm and 5 mm, respectively, to ensure measurement accuracy. In literature (Meaney et al., 2016), Meaney compared the sensing depths of probes with diameters of 18 mm and 21 mm and the study reported that the effective sensing depths of 18 mm and 21 mm diameter probes were 1.84 mm and 2.75 mm, respectively. Another comparative study was carried out in literature (Gioia et al., 2020) for probes with diameters of 2.20, 9.5, and 19 mm, and the results showed that the sensing radius scales linearly with the inner diameter of the outer conductor. The effect of the inner conductor on the sensing radius is greater than the effect of the insulator on the sensing radius. In 2022, Aydinalp et al. (2022) conducted simulation experiments to investigate the sensing depth of probes with three different diameters (0.5, 0.9, and 2.20 mm), and verified the results of the simulations with actual measurements using a commercial probe with a diameter of 2.20 mm. Although the effect of probe aperture size on the depth of sensing has been studied to some extent in the literature, the sensing radius of different probe apertures, especially small aperture probes, has not been fully explored. Defining the sensing radius for small aperture probe sizes, and thus using the appropriate probe size, is important for accurate measurements of the dielectric properties of multilayered tissues, such as breast cancer tissue. In addition, an accurate and reproducible measurement system and protocol to quantify the sensing radius needs to be established and must be validated by measuring specific tissue types to enable the practical application of open coaxial probes, mitigating the associated errors introduced by equipment and heterogeneous tissues.

Meanwhile, in dielectric measurement research where precision displacement control is essential, existing approaches include manual translation stages (Yuen et al., 1990), piezoelectric actuators (Ling, et al., 2021), and stepper motor-driven platforms (Zhang, et al., 2021). Each method presents limitations: conventional stages are bulky with poor repeatability; piezoelectric systems offer high precision but suffer from limited travel (<100 μm), high cost, and complex control requirements; motorized platforms introduce mechanical vibration, positioning hysteresis, and electromagnetic interference in sensitive measurements. To the best of our knowledge, no prior study has reported the rigid integration of high-precision ball linear slides with micrometer heads for coaxial probe sensing range characterization, A dedicated comparison table (Table 1) was created for technical benchmarking.

**TABLE 1:** Comparative analysis of precision positioning systems for dielectric sensing range characterization.

Positioning method	Precision	Repeatability	Max travel	Cost	Key limitations
Manual Translation Stages (Yuen et al., 1990)	∼0.1 mm	Low	>50 mm	Low	Bulky design, operator-dependent errors, poor repeatability
Piezoelectric Actuators (Ling, et al., 2021)	<0.001 mm	High	<0.1 mm	Very High	Limited travel range, complex control algorithms, significant EMI, high cost
Stepper Motor-Driven Systems (Zhang, et al., 2021)	0.01–0.05 mm	High	>100 mm	High	Mechanical vibration, positioning hysteresis, electromagnetic interference (EMI)
Caliper-Based Methods (Common practice)	0.1 mm	Low	>100 mm	Low	Manual measurement errors, contact force variations, no axial alignment control
Proposed System (Sliding rail + micrometer)	0.01 mm	High	>100 mm	Low	Limited to linear measurements (single axis)

Our system’s first key innovation lies in its sub-micron positioning capability (0.01 mm resolution), achieved through zero-backlash linear guides and high-resolution micrometer reading. This represents a 10× improvement over caliper-based methods (0.1 mm) commonly used in sensing depth measurements (Liu et al., 2020). The second major advancement is exceptional measurement repeatability (±0.05 mm), enabled by the slide rail’s superior straightness and rigidity which eliminate lateral wobble, contact-force variations, and environmental vibrations. This stability is critical for long-term monitoring and repeated verification in dielectric studies. Compared to high-cost solutions like precision motorized stages or piezoelectric systems, our approach maintains comparable accuracy while offering simplified operation and significantly reduced cost. Against similarly priced manual stages, it delivers substantially improved precision and stability. The compact, fixture-compatible design further provides unique advantages for coaxial probe measurements in confined spaces.

In this context, the present work aims to systematically examine the sensing radius and depth performance of two prevalent small-aperture coaxial probes developed for breast cancer detection applications. Defining the depth of sensing and radius of the probe can help to minimize errors in the characterization of dielectric properties. Unlike previously reported studies, this study establishes a reliable and reproducible measurement protocol for quantifying the sensing radius of open coaxial probes. The rest of the paper is organized as follows: Section 2, background. In Section 3, the materials and methods including the experimental setup, sample configuration and measurement protocol are explained in detail. Section 4 presents the experimental results. Section 5 discusses the findings in detail.

## 2 Theoretical background

**FIGURE 1:**
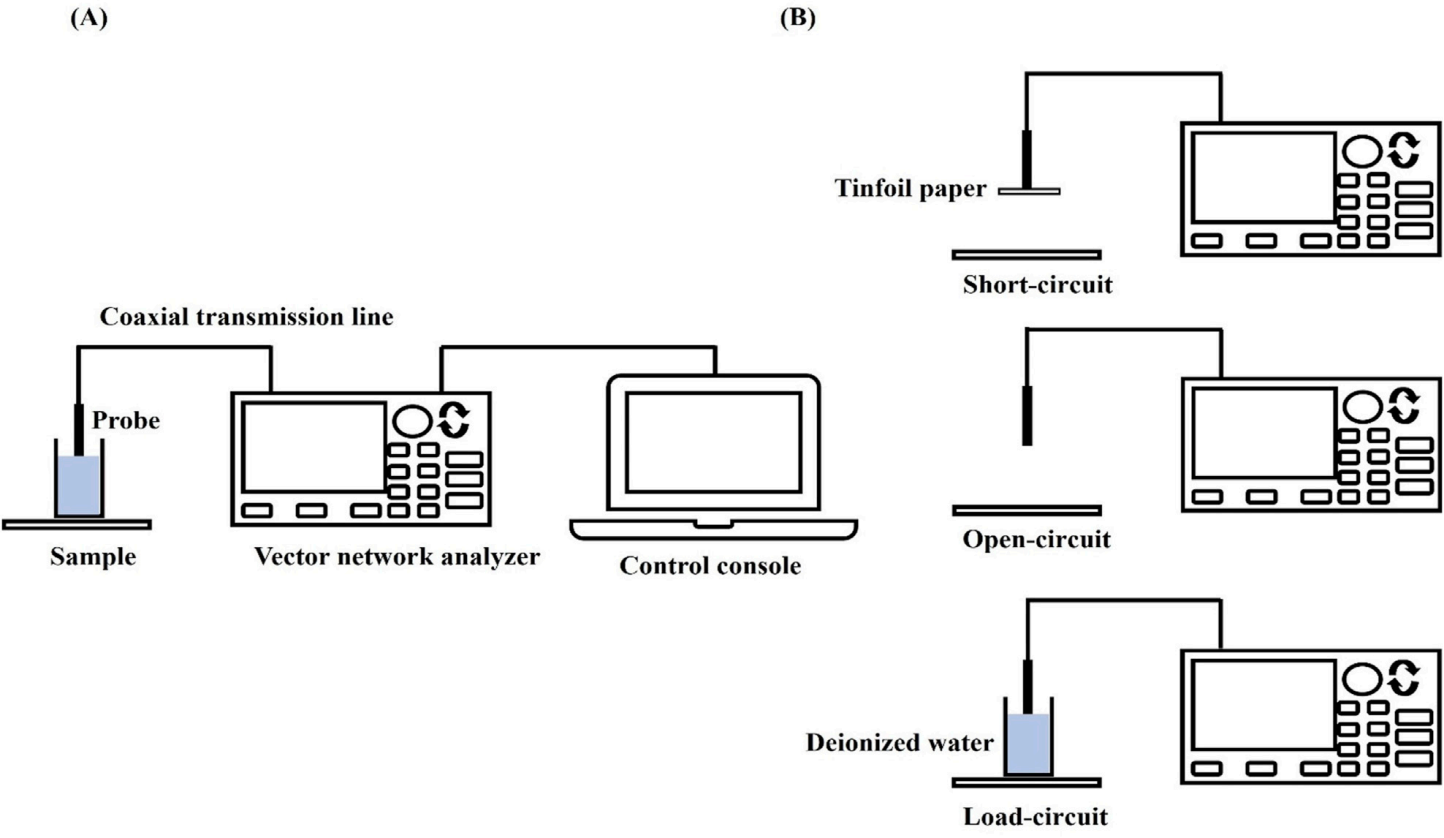
VNA measurement settings and calibration procedure: **(A)** VNA measurement setup; **(B)** Calibration flow.

According to the transmission line theory, electromagnetic waves can not only propagate in the wireless free space, but also can be guided to propagate using a closed electromagnetic system (e.g., waveguide system), and the device that guides the propagation of electromagnetic waves is called a transmission line, e.g., the open-ended coaxial probe used in the present experiments belongs to a kind of transmission line (shown in Figure 1A). When biological tissues are in direct contact with the coaxial probe, electromagnetic wave reflection occurs at the probe terminus due to impedance mismatch between the probe termination and the measured tissue. Therefore, the dielectric properties of the tissue under test can be calculated from the reflection coefficient measured by a vector network analyzer, where the relationship between the tissue’s complex dielectric properties and the reflection coefficient is given by Equation 1:

$$\varepsilon^{*}=\varepsilon_{r}^{\prime }-j\frac{\sigma }{\omega \varepsilon_{0}}=\frac{A_{1}\rho_{m}-A_{2}}{A_{3}-\rho_{m}}$$ (1)

where $\varepsilon^{*}$ represents the dielectric properties of the tissue to be measured, $\varepsilon_{r}^{\prime }$ is the relative permittivity of the tissue to be measured, $\rho_{m}$ is the reflection coefficient obtained from the VNA measurements, *A1*, *A2* and *A3* can be determined by the three-parameter method as described in Equations 2–4 (Kraszewski et al., 1983):

$$\rho_{1}=A_{3}$$ (2)

$$\rho_{2}=\frac{A_{2}+A_{3}}{A_{1}+1}$$ (3)

$$\rho_{3}=\frac{A_{2}+A_{3}\left(\varepsilon_{r^{\prime }}^{\prime }-j\frac{\sigma_{s}}{\omega \varepsilon_{0}}\right)}{A_{1}+\left(\varepsilon_{r^{\prime }}^{\prime }-j\frac{\sigma_{s}}{\omega \varepsilon_{0}}\right)}$$ (4)

where $\rho_{1},\rho_{2}$ and $\rho_{3}$ represent the reflection coefficients of the short-circuit, open-circuit, and standard liquid loads, respectively, $\varepsilon_{r}^{\prime },$ and $\sigma_{s}$ represent the relative permittivity and conductivity of the standard liquids to be tested, respectively (shown in Figure 1B).

**FIGURE 2:**
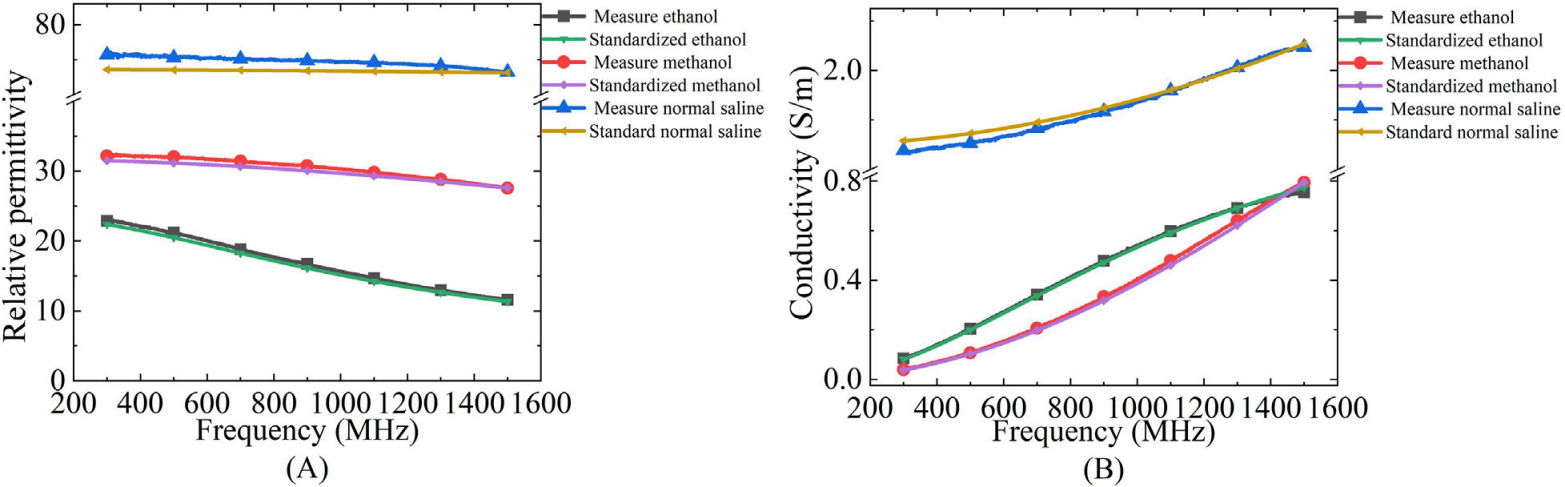
Comparison of dielectric properties of known materials (ethanol, methanol and saline) calculated from Debye parameters obtained from literature and measured by experimental setup: **(A)** Relative permittivity; **(B)** Conductivity.

The measurement system uses a dedicated dielectric thin open coaxial probe (Suzhou TaiLai Microwave Technology Co., Ltd.) connected to a VNA (Agilent E5063A, 1.5 GHz) to measure the reflection coefficients of the scanned samples to be measured. In this study, we validated the calibration method for VNA system measurements using three materials with known values of dielectric properties: ethanol, methanol and saline (154 mol/L). The calibrated measurements were compared with the dielectric properties calculated in the literature based on the Debye parameters (Gregory and Clarke, 2012). The measured and calculated dielectric properties are shown in Figure 2. Furthermore, based on the Debye parameters reported in the literature, Table 2 presents the maximum deviations between measured and calculated values at corresponding frequency points.

**TABLE 2:** Difference between the measured values and the maximum dielectric properties calculated by the Debye model for the known materials used for system validation.

Materials	Different $\varepsilon_{r}^{\prime }$	Frequency (MHz)	Different σ (S/m)	Frequency (MHz)
Ethanol	0.65	882	0.01	1,500
Methanol	0.97	328	0.02	1,217
Saline	2.50	310	0.05	318

## 3 Materials and methods

This section details the dielectric properties measurement system, the sensing depth and sensing radius measurement model, the preparation of the bilayer sample structure and the sensing volume measurement scheme used.

### 3.1 Dielectric measurement system

**FIGURE 3:**
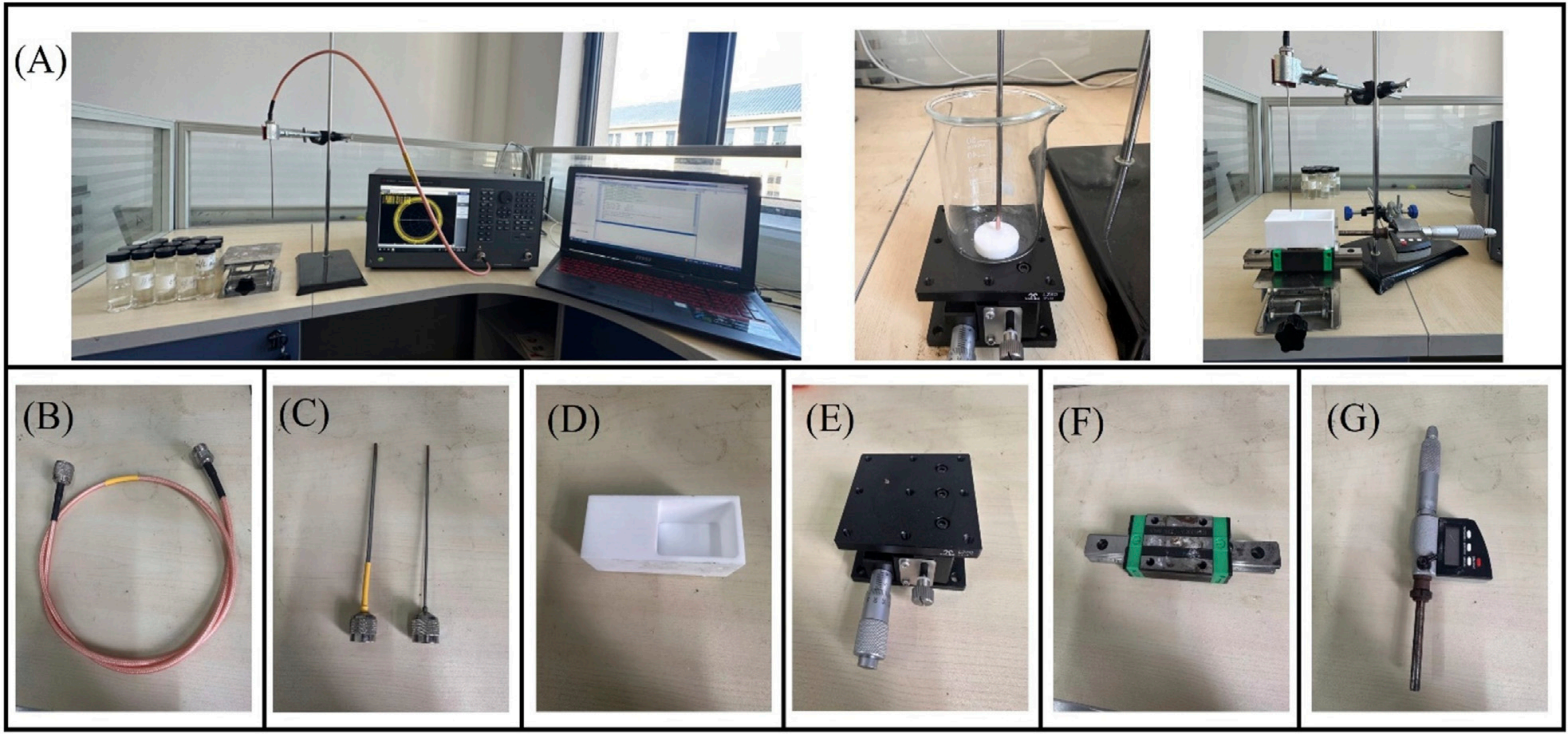
Experimental platform for effective sensing volume: **(A)** Schematic diagram of the effective sensing volume measurement; **(B)** Flexible coaxial wire; **(C)** Open-end coaxial probe; **(D)** Teflon water tank; **(E)** Digital lift table; **(F)** Slide rail; **(G)** Digital micrometer.

In this study we used a VNA to measure the reflection coefficient from the probe. To eliminate temperature drift errors caused by equipment and cable connections, the VNA was switched on 2 h before the measurement. To investigate the frequency-dependent characteristics of the probe’s sensing volume, measurements were conducted at 200 MHz intervals across the frequency spectrum from 300 MHz to 1.5 GHz. The experimental setup incorporated a high-precision screw-adjustable positioning stage (YanTuo Precision Factory, 0–10 mm range, 0.01 mm digital resolution) in conjunction with a digital micrometer (Dongguan SanLiang Measurement Co. Ltd., 0–150 mm range, 0.01 mm resolution) for accurate positioning and documentation of the bilayer sample configuration during measurements. The experimental setup is shown in Figure 3. The current study utilizes two distinct open-ended coaxial probes featuring small aperture sizes of 2.20 mm and 3.58 mm in diameter (supplied by Suzhou TaiLai Microwave Technology Co., Ltd.). These probes are characterized by a dual-conductor structure with an intermediate dielectric layer, and their dimensional parameters are summarized in Table 3.

**TABLE 3:** Dimensions of the two coaxial probe models.

Probe model	Inner conductor (mm)	Insulator (mm)	Outer conductor (mm)
G1	0.51	1.68	2.20
G2	0.92	2.98	3.58

The 2.20 mm diameter represents a standard commercial size for superficial dielectric measurements (Aydinalp et al., 2022). This dimension was specifically selected to satisfy clinical requirements for shallow tissue assessment, since early-stage tumor evaluation necessitates measurements at ∼0.5 mm depth for detection of epidermal/dermal water content variations. In parallel, the 3.58 mm probe was incorporated to enable systematic examination of geometric scaling effects on sensing depth and radius. The resulting 1.63:1 diameter ratio permits clinically meaningful comparisons while maintaining the study’s focus on early disease detection.

### 3.2 Structure of the sensing depth and sensing radius measurement model

**FIGURE 4:**
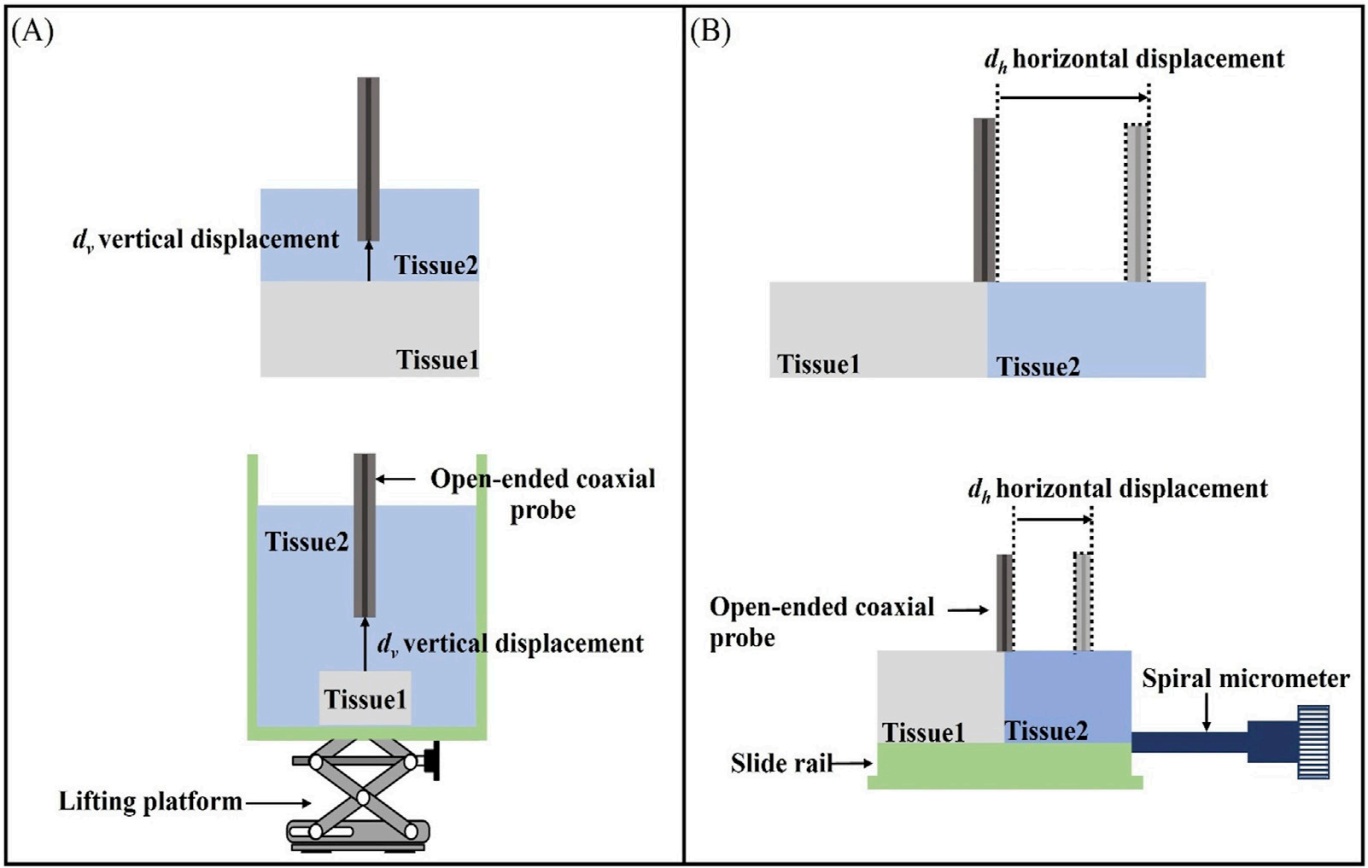
Schematic representation of the effective sensing volume: **(A)** Schematic representation of the effective sensing depth vertical displacement $d_{v}$ ; **(B)** Schematic representation of the effective sensing radius horizontal displacement $d_{h}$ .

In this paper, a two-layer structure model is employed to represent the heterogeneous tissue component composition (Figure 4). When measuring a homogeneous Tissue 1 with dielectric properties $\varepsilon_{1}$ , $\sigma_{1}$ , an adjacent Tissue 2 with distinct dielectric properties $\varepsilon_{2}$ , $\sigma_{2}$ is presented. Taking the centre of the coaxial probe cross-section as the starting point of the distance, the distance moved along the vertical direction is $d_{v}$ ; the distance moved along the horizontal direction is $d_{h}$ .

### 3.3 Sample configuration

This experiment represents the heterogeneous tissue composition by using a bilayer structure consisting of Teflon (Teflon, $\varepsilon^{\prime }\approx 2.1$ ) and liquid under test (LUT) (Aleem et al., 2021). The Teflon cube was set as Tissue 1, and the liquid as Tissue 2 (Figure 4). Three distinct test liquids with varying relative permittivity values were selected based on their dielectric contrast with Teflon and Tissue 2, as detailed in Table 4. Ethanol was chosen to represent materials with low relative permittivity, methanol for intermediate values, and deionized water for high relative permittivity. This selection provides comprehensive coverage across a wide range of dielectric values, ensuring representative characterization. The temperature of the LUT was measured prior to each measurement. Furthermore, dimethyl sulfoxide (DMSO) and proportional sugar-salt mixed solutions were employed to simulate the relative permittivity of porcine muscle tissue and breast cancer tissue (Duan et al., 2014), while Teflon was utilized to mimic the dielectric properties of porcine adipose tissue and breast fat tissue during measurements.

**TABLE 4:** Relative permittivity of LUT at 300 MHz, 20 °C.

Sample	$\varepsilon^{\prime }$
Teflon	2.1
Ethanol	22.7
Methanol	33.3
Deionized water	80.2
DMSO	47.0
Sugar-salt mixed solution	60.0

DMSO was selected as a muscle tissue phantom due to its dielectric properties at 300 MHz, a frequency commonly employed in microwave ablation applications. According to Gabriel et al. (1996), DMSO (ε′ ≈ 45, σ ≈ 0.8 S/m) demonstrates close approximation to human skeletal muscle (ε′ = 50–55, σ = 0.7–0.9 S/m). This correspondence arises from DMSO’s high dielectric constant (ε′ ≈ 46.7), which effectively replicates the polar aqueous environment characteristic of muscle tissue (porcine).

For breast cancer tissue simulation, a sugar-salt mixture was implemented through a dual-path approach involving ionic conductivity enhancement and dielectric constant elevation. The ionic conductivity is modulated through NaCl dissociation, providing tunable conductivity (σ ∝ [NaCl]) as demonstrated in liver phantoms by Peyman et al., achieving linear tunability within σ ≈ 0.5–1.5 S/m (Peyman et al., 2015). Simultaneously, sucrose disrupts the hydrogen-bond network of free water, increasing the dielectric constant by 10%–15% compared to pure salt solutions, thereby simulating the high water content (>70%) typical of tumor tissue. At 300 MHz, this sugar-salt mixture (ε′ ≈ 60, σ ≈ 1.2 S/m) shows excellent agreement with standard values for malignant breast tissue (ε′ = 55–65, σ = 1.1–1.3 S/m for ∼20% fat content), as established by *ex vivo* measurements in the Lazebnik database (Lazebnik et al., 2007).

### 3.4 Measurement of sensing depth and sensing radius

For the sensing depth measurement, we made a measurement model as shown in Figure 4A. The open-end coaxial probe was inserted upwards in the measurement cell and was in contact with Tissue 1 (Teflon) fixed in the measurement cell. The other end of the probe is connected to the VNA via a flexible coaxial cable, which is held in place by tape at several locations along its length, thus reducing measurement errors due to cable movement during the measurement. The measurement chamber was filled with LUT representing Tissue 2, thereby establishing a stratified model configuration of open-ended coaxial probe/Tissue 2/Tissue 1, which effectively simulates the vertical stratification of biological tissue samples. The measurement protocol was initiated upon establishing complete contact between the probe and Tissue 1. The vertical positioning stage was then precisely controlled to displace Tissue 1 downward in 0.05 mm increments along the vertical axis, with dielectric property measurements systematically recorded at each displacement interval.

Figure 4B illustrates the layered model used in this study for measuring the effective sensing radius of the probe. The layered model in the horizontal direction was constructed by filling the LUT representing Tissue 2 (as listed in Table 2) into a Teflon-printed tank designed to simulate Tissue 1. The probe was placed at the center of the two tissues as a measurement start point and the sink was tightly connected to a sliding rail. The displacement of a sliding rail was precisely controlled by a spiral micrometer to change the position of the probe relative to the layered interface. The spiral micrometer was moved in increments of 0.05 mm and the dielectric properties were measured after each movement. The process was repeated by replacing the probe with different diameters and the LUT, while experimental results were systematically recorded.

All sensing depth and radius evaluations were performed with comprehensive repeat measurements. For each experimental condition - comprising seven media types (five tissue-mimicking phantoms and two biological tissues) tested with two probe sizes - five independent replicates were conducted.

To minimize operator-related bias, we implemented a comprehensive quality control protocol. Sample testing sequences were randomized through computer-generated schedules to eliminate ordering effects. While operators necessarily maintained awareness of sample types during real-time impedance monitoring, all measurements were performed independently by two certified technicians (S. Chen and Z. Lu) adhering to standardized operating procedures with daily role alternation. Crucially, objective quantification parameters including depth and radius measurements were determined automatically using validated image processing algorithms to remove subjective interpretation.

### 3.5 Biological tissue measurements

**FIGURE 5:**
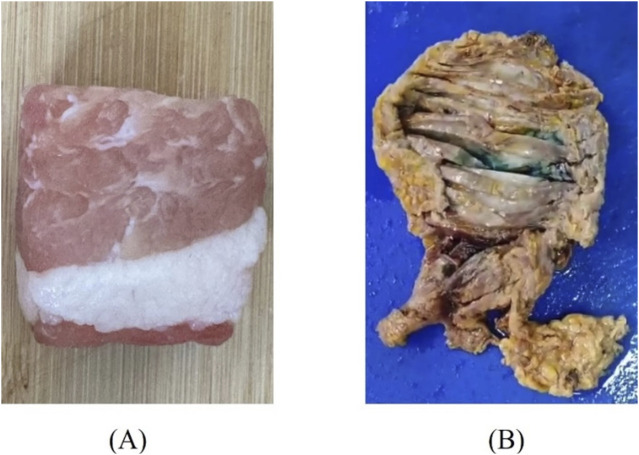
Diagram of tissue samples: **(A)** Porcine fat-muscle tissue; **(B)** Human breast cancer tissue.

Tissues with distinct layered structures, including porcine fat-muscle tissue and human breast cancer tissue, were selected for comparative validation of the aforementioned layered model (Figure 5). Two different sizes of probes, 2.20 mm and 3.58 mm were employed. Porcine tissue was selected due to its well-defined heterogeneous structure, which consists of two easily distinguishable components—fat and muscle tissues. Meanwhile, breast cancer tissue was chosen because of the significant contrast in dielectric properties between cancerous and adipose tissues, facilitating clear differentiation.

Fresh porcine tissues, immediately after excision, were obtained from a local slaughterhouse, stored in sealed plastic bags, and transported to the laboratory with ice packs. Tissue dehydration was minimized by limiting the duration of each sample’s exposure to air. The human breast cancer tissue samples used were taken from patients diagnosed with breast cancer at a local hospital. After diagnosis by the pathologist, they were immersed in formalin liquid and stored in a refrigerator at −4 °C. During the measurement, it was necessary to wait for the tissues to return to normal temperature and then rinse them several times using water to remove the formalin solution present on the surface of the tissues in order to obtain reliable measurements. Prior to the sensing volume measurement, the dielectric properties of biological materials were measured, with each sample subjected to three repeated measurements. This experiment has been approved by the local ethics committee of Ganzhou People’s Hospital (code PJB2024-011-01 Approval date 2024-11-19).

To validate the effective sensing depth of the probe, a two-layer structure was employed, with the muscle layer as the first layer (Tissue 2) and the fat layer (Tissue 1) as the second layer. Dielectric property measurements were conducted at four different locations using probes of two distinct sizes. For breast cancer tissue, the effective sensing depth of the probe was measured using a two-layer structure, with cancerous tissue as the first layer (Tissue 2) and mammary adipose tissue as the second layer (Tissue 1). The effective sensing radius of the probe was verified in the same way as for the LUT measurements described above.

Furthermore, the study incorporated a single-blind design wherein raw data were entered blindly by an independent research assistant (G. Zhao) who had no involvement in the measurement process. Operator roles were strictly limited to sample positioning and initiation of predefined measurement protocols, ensuring complete segregation between data collection and analysis phases.

### 3.6 Data collection

Given that the dielectric property differences between normal tissue and cancerous tumor tissue typically exceed 10%, the sensing depth in this experiment was defined as the distance moved by the probe when the measured value reached 90% of the dielectric property parameters of Tissue 2 (Hagl et al., 2003). Similar to the sensing depth, the sensing radius was defined as the horizontal distance moved by the coaxial probe when the measured value reached 90% of the relative dielectric permittivity of Tissue 2.

Since the dielectric properties of the pure material are known, the above percentage change can be calculated mathematically. On the other hand, the dielectric property measurements obtained from spatial position movements may not align with the expected percentage change. Therefore, an error range was defined to account for such discrepancies. If the measured dielectric properties change at a specific distance fall within 3% of the expected percentage change, that distance is recorded (Aydinalp et al., 2021). Otherwise, If the measured value at a specific spiral micrometer displacement exceeds the expected percentage (>93%), the corresponding micrometer displacement with a measured value below the expected percentage (<87%) is then selected. The average between the two distances is chosen as the distance corresponding to the expected change in dielectric properties (Aydinalp et al., 2021).

The sensing volume data from seven representative frequencies (300, 500, 700, 900, 1,100, 1,300, and 1,500 MHz) for three tissue-mimicking liquids (water, methanol, and ethanol) were subjected to one-way ANOVA to quantitatively assess stability across the band. The frequency-stability analysis was not performed for the DMSO and sugar-salt solutions, as their dielectric properties were characterized exclusively at a single frequency (300 MHz), at which they demonstrated close matching with target tissues.

## 4 Results

This section compares the variations in the effective sensing volume of two differently sized probes using ethanol, methanol, and deionized water as LUT. Subsequently, the variations in the effective sensing volume of the probes with respect to measurement frequency are reported. Finally, the sensing depth and sensing radius measurement results are validated using porcine tissue and breast cancer tissue.

### 4.1 LUT sensing volume measurements

**FIGURE 6:**
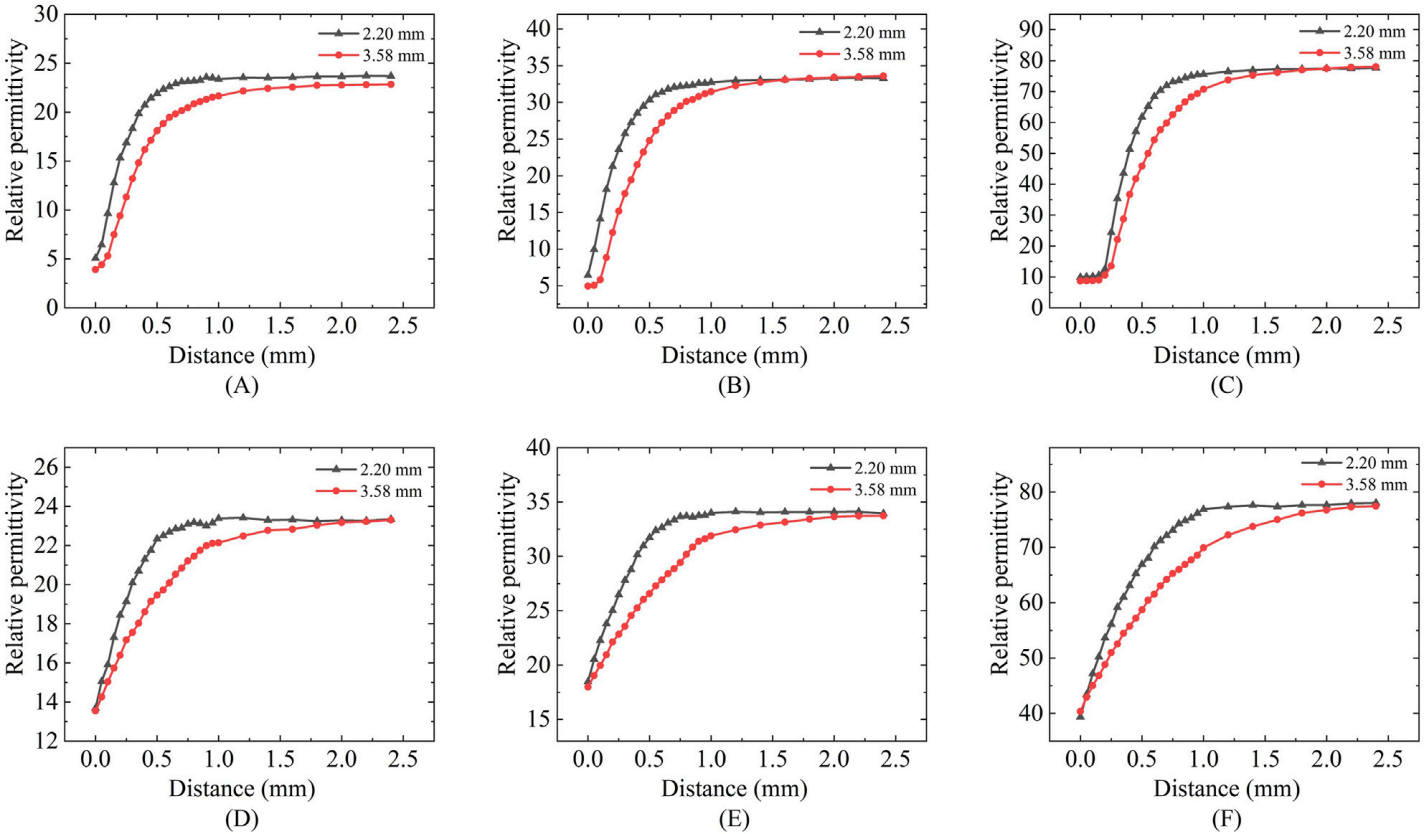
Effective sensing depth and radius of the 2.20 mm and 3.58 mm probes: **(A,D)** Ethanol solution; **(B,E)** Methanol solution; **(C,F)** Deionized water solution.

Figure 6 presents the results of sensing depth and sensing radius measurements performed using a two-layer model at a measurement frequency of 300 MHz. Figures 6A–C shows the effective depth of sensing for ethanol, methanol, and deionized water measured with two different sizes of probes. We can find that the relative permittivity changes rapidly with the increase of the vertical displacement distance of the Teflon in the first part of the section, whereas the relative permittivity obtained from the measurements in the second half of the section varies slowly and is close to that of the actual measured liquid. Different sizes of probes have different depths of sensing, and the effective depth of sensing of the probe increases as the probe size increases (Table 5).

**TABLE 5:** Sensing volume for different probe sizes.

Sample	2.20 (mm)				3.58 (mm)			
	$d_{v}$ (mm)	Standard deviation	$d_{h}$ (mm)	Standard deviation	$d_{v}$ (mm)	Standard deviation	$d_{h}$ (mm)	Standard deviation
Ethanol	0.44	0.04	0.36	0.02	0.75	0.03	0.71	0.04
Methanol	0.50	0.03	0.42	0.02	0.81	0.04	0.81	0.04
Deionized water	0.62	0.02	0.63	0.02	0.98	0.02	0.99	0.02

Figures 6D–F shows the effective sensing radius of ethanol, methanol, and deionized water measured with two different sizes of probes; a similar process of change to the vertical displacement can be observed, where the change in the relative permittivity changes from fast to slow as the distance travelled increases. Different sizes of probes have different sensing radius, and the effective sensing radius of the probes increases as the probe size increases (Table 5).

Table 5 summarizes the effective sensing depth and radius of two differently sized probes when three different LUT were measured. Taking the 2.20 mm diameter probe as an example, the effective sensing depth is 0.44 mm for ethanol, 0.5 mm for methanol, and 0.62 mm for deionized water; The effective sensing radius is 0.36 mm for ethanol, 0.42 mm for methanol, and 0.63 mm for deionized water. This suggests that tissues with different contrasts have different sensing volume.

**FIGURE 7:**
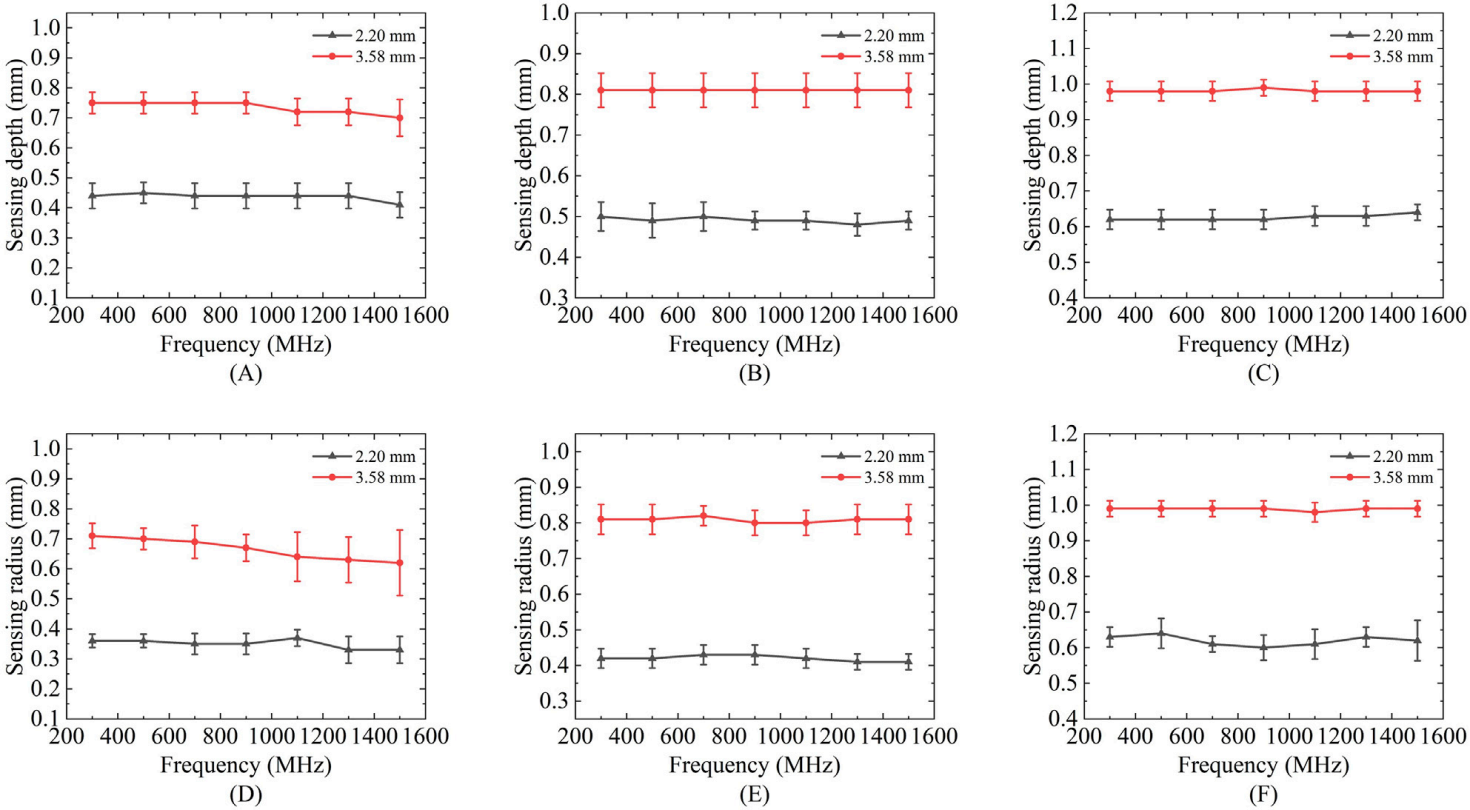
Variation of sensing depth and radius with frequency for 2.20 mm and 3.58 mm probes: **(A,D)** Ethanol solution; **(B,E)** Methanol solution; **(C,F)** Deionized water solution.

Figure 7 presents the variations in the effective sensing volume of the probe across the frequency range of 300 MHz to 1.5 GHz for different solutions. Figures 7A–C show the changes in the sensing depth, while Figures 7D–F display the changes in the sensing radius. With the increase of the measurement frequency, the effective sensing volume of the probe is relatively stable, and does not change significantly with the frequency.

**FIGURE 8:**
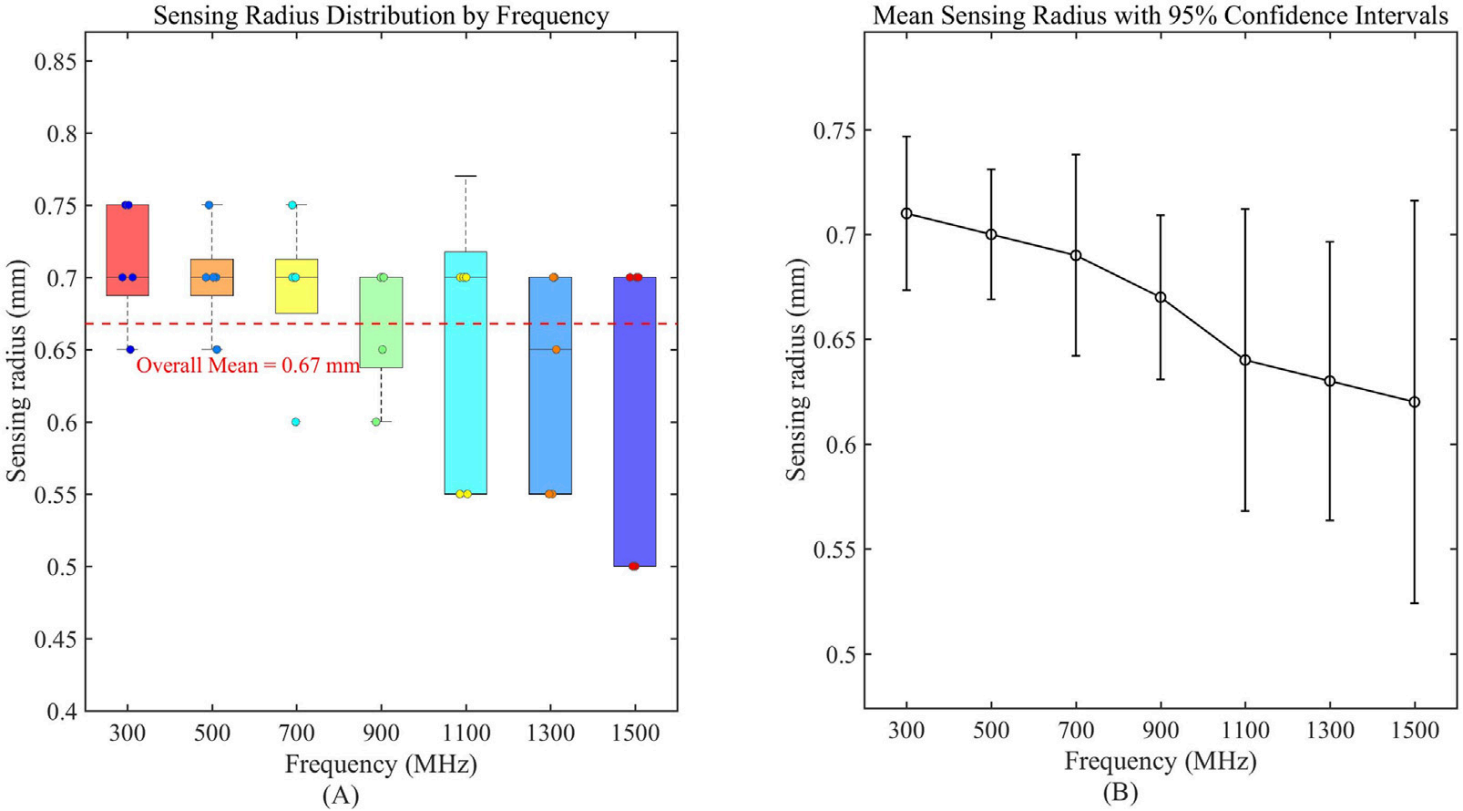
Frequency-dependent characteristics of sensing volume distribution. **(A)** Boxplot analysis of sensing radius distribution across measured frequencies; **(B)** Mean sensing radius with 95% confidence intervals, demonstrating stability across the operational frequency range.

Results showed no statistically significant difference across frequencies for any of these liquids (p > 0.05), confirming volumetric stability within this range. For the comprehensive demonstration of these findings, a new boxplot (Figures 8A,B) has been included to compare sensing volume distributions across key frequencies. Representative ethanol data (probe radius 3.58 mm) were selected for visualization due to space considerations, while complete statistical analysis including F-statistics and p-values for all tested liquids is provided in Table 6. Collectively, these results serve to reinforce the evidence for sensing volume stability throughout the operational frequency band.

**TABLE 6:** Frequency stability analysis of sensing volume (one-way ANOVA).

Sample	2.20 (mm)				3.58 (mm)			
	Sensing depth		Sensing radius		Sensing depth		Sensing radius	
	F-statistic	p-value	F-statistic	p-value	F-statistic	p-value	F-statistic	p-value
Ethanol	0.4681	0.8260	0.9899	0.4510	1.1765	0.3469	1.1836	0.3433
Methanol	0.2564	0.9524	0.4912	0.8093	0.0038	1.0000	0.1626	0.9846
Deionized water	0.4333	0.8503	0.7000	0.6519	0.1000	0.9958	0.1333	0.9908

### 4.2 Biological tissue measurements

**FIGURE 9:**
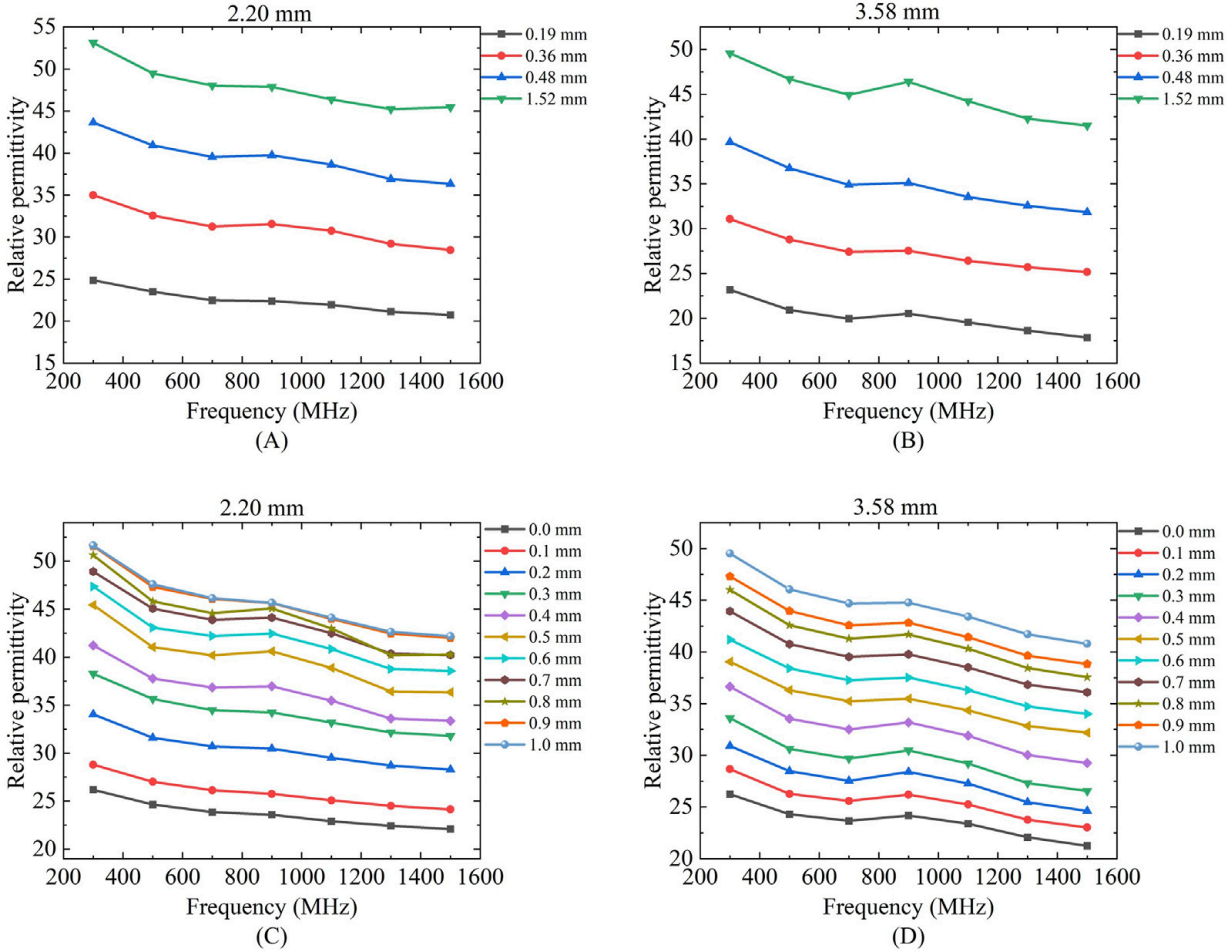
Variation of effective sensing depth and sensing radius with frequency for two probes for porcine tissue: Figures **(A,C)** Porcine tissue measured by 2.20 mm probe; **(B,D)** Porcine tissue measured by 3.58 mm probe.

Figure 9 presents the measurement results of the sensing depth and radius for porcine tissue using two differently sized probes. Due to the difficulty in accurately controlling the thickness of muscle tissue, the effective sensing depth of the probe was measured at four selected locations with muscle layer thicknesses ranging from thin to thick: 0.19, 0.36, 0.48, and 1.52 mm. According to the two-layer model measurement results, the relative permittivity measured for the muscle layer with a thickness of 0.19 mm is significantly lower than that measured for the other three muscle layer thicknesses. For the four different thicknesses of the muscle layer, the two different sizes of the probes can produce different measurements, and the relative permittivity measured by the diameter of the 2.20 mm probe at 300 MHz are 24.84, 34.99, 43.65, and 53.12, respectively and the relative permittivity of the four different muscle layer thicknesses measured by the probe with a diameter of 3.58 mm at 300 MHz are 23.17, 31.07, 39.65, and 49.56, respectively, the results indicate that the larger the probe size, the relative permittivity were smaller, which verified that the larger size of the probe had a deeper effective sensing depth.

As shown in Figures 9C,D which present the effective sensing radius measurements of the 2.20 mm and 3.58 mm probes for porcine tissue, it can be observed that the measured relative permittivity gradually increases and approaches the relative permittivity of muscle tissue as the moving distance increases. The two probes of different sizes exhibit distinct effective sensing radius. At a frequency of 300 MHz, the effective sensing radius of porcine tissue measured by the 2.20 mm coaxial probe is 0.54 mm, while that measured by the 3.58 mm coaxial probe is 0.72 mm.

**FIGURE 10:**
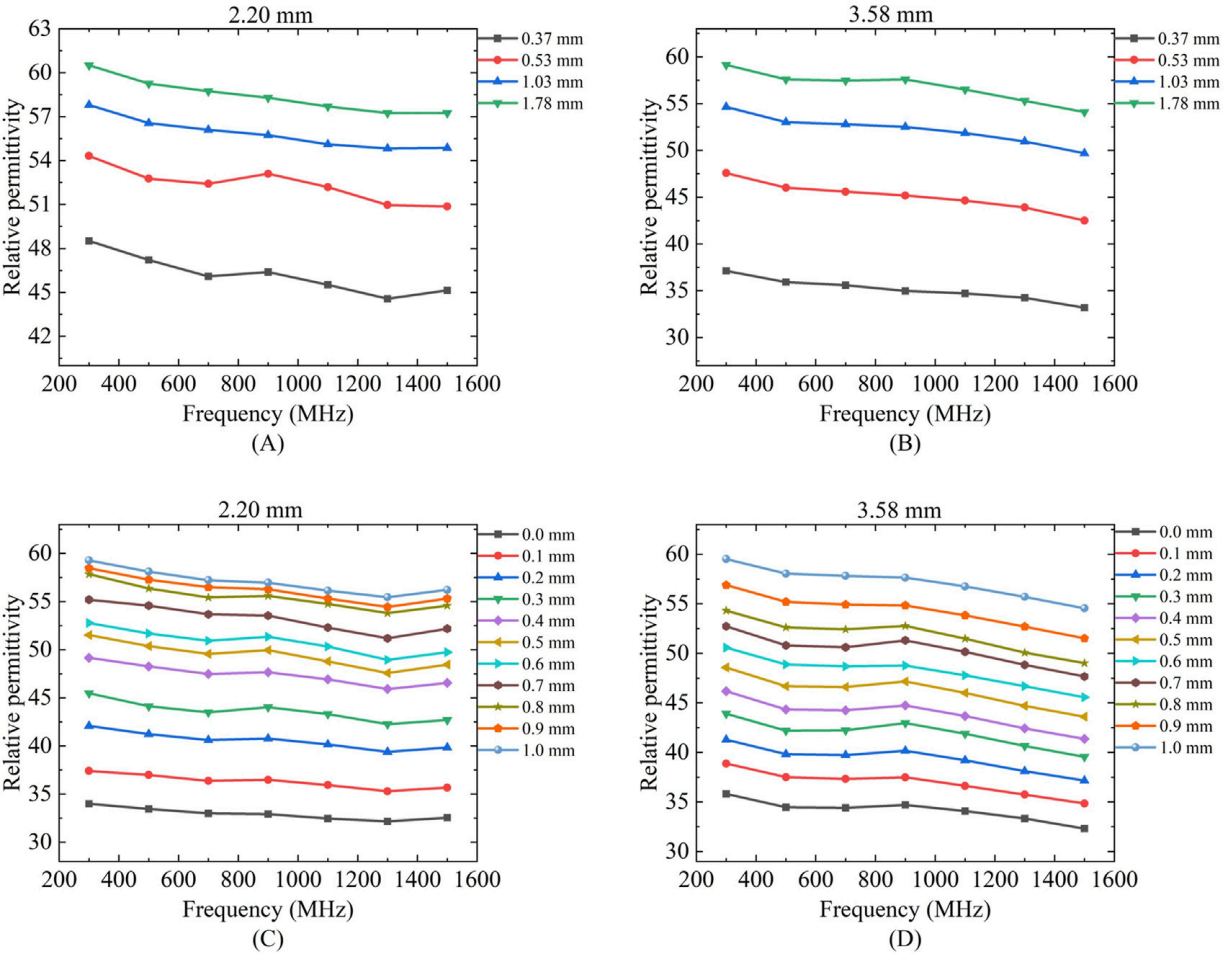
Variation of effective sensing depth and sensing radius with frequency for two probes for breast tissue: Figures **(A,C)** Human breast tissue measured using 2.20 mm probe; **(B,D)** Human breast tissue measured using 3.58 mm probe.

Figure 10 presents the actual measurement results of the effective sensing depth and radius for breast cancer tissue using probes of different sizes. For the measurement of the effective sensing depth of breast cancer tissue, the same method as that used for porcine tissue was applied, with measurements taken at four selected locations with tissue thicknesses ranging from thin to thick: 0.37, 0.53, 1.03, and 1.78 mm. According to the layered model measurement results, the measured values for the breast cancer tissue layer with a thickness of 0.37 mm are expected to be significantly lower than those for the other three breast cancer tissue layer thicknesses. The measurement results of the 2.20 mm and 3.58 mm probes are shown in Figures 10A,B. For the four different thicknesses of breast cancer tissues, two different sizes of probes can produce different measurement results, and the relative permittivity of the four different thicknesses of breast cancers measured by a probe with a diameter of 2.20 mm at 300 MHz is 48.51, 54.32, 57.79, and 60.50, respectively. The relative permittivity of four different breast cancer thicknesses measured by a probe with a diameter of 3.58 mm at 300 MHz are 37.12, 47.58, 54.65, 59.13, respectively. The larger the size of the probe, the smaller the relative permittivity measured, which also proves that the larger size of the probe has a deeper effective sensing depth.

Figures 10C,D shows the effective sensing radius of breast cancer tissues with 2.20 mm and 3.58 mm probes, we can find that with the increase of travelling distance, the relative permittivity of the measurements gradually increases and keeps approaching to that of the breast cancer tissues. The two different sizes of the probes have different effective sensing radius, the effective sensing radius of breast cancer tissues is 0.6 mm with the coaxial probe of the size of 2.20 mm and 0.76 mm with the coaxial probe of the size of 3.58 mm at a frequency of 300 MHz.

### 4.3 Validation of biological tissue measurements

**FIGURE 11:**
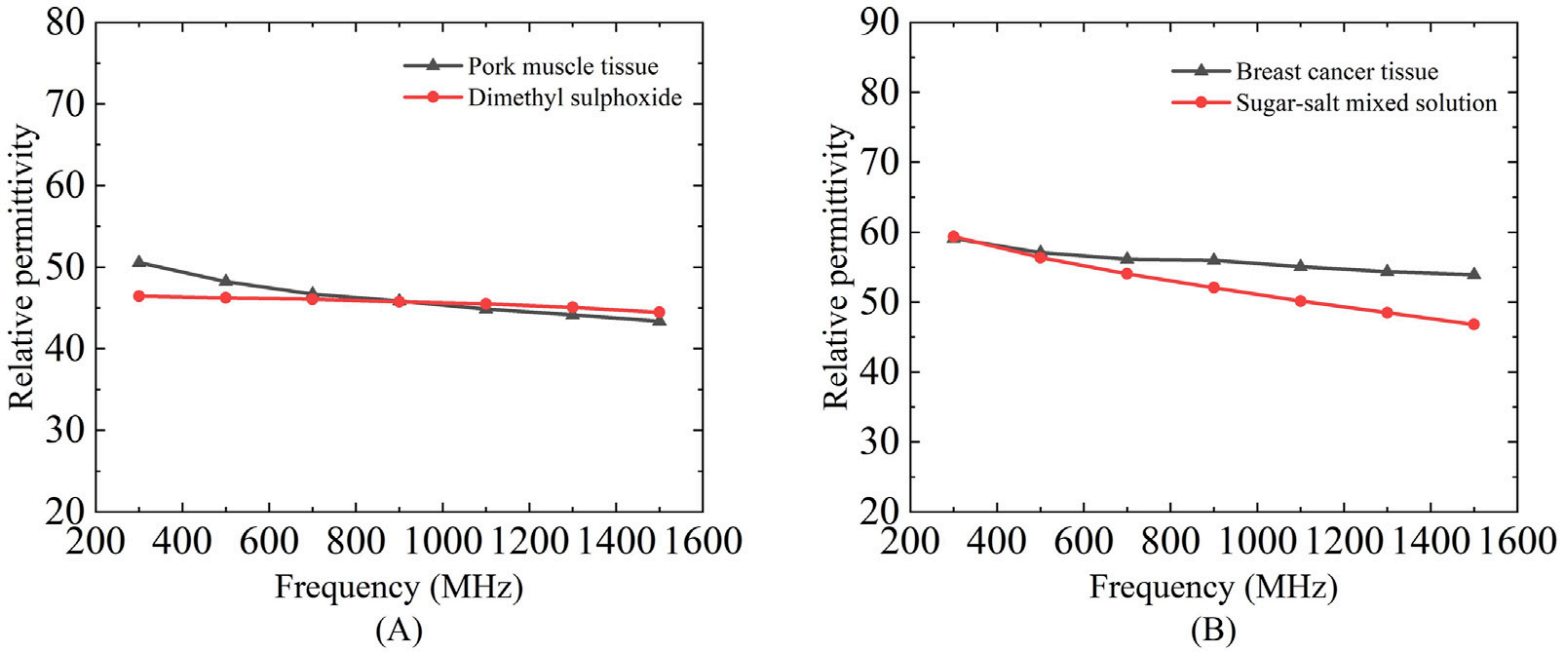
Dielectric properties of a mixed solution of dimethyl sulfoxide and proportional sugar salts selected to mimic porcine muscle tissue and breast cancer tissue: **(A)** Relative permittivity of dimethyl sulfoxide and pork muscle tissue; **(B)** Relative permittivity of sugar-salts mixed solution and breast cancer tissue.

Due to the difficulty in precisely controlling the actual thickness of tissue slices, dimethyl sulfoxide (DMSO) and a proportional sugar-salt mixture were used to mimic porcine muscle tissue and breast cancer tissue, respectively, in terms of relative permittivity, in order to investigate the effective sensing depth of these two tissues. Teflon was employed to simulate porcine adipose tissue and mammary adipose tissue for the measurements (Figure 11).

**FIGURE 12:**
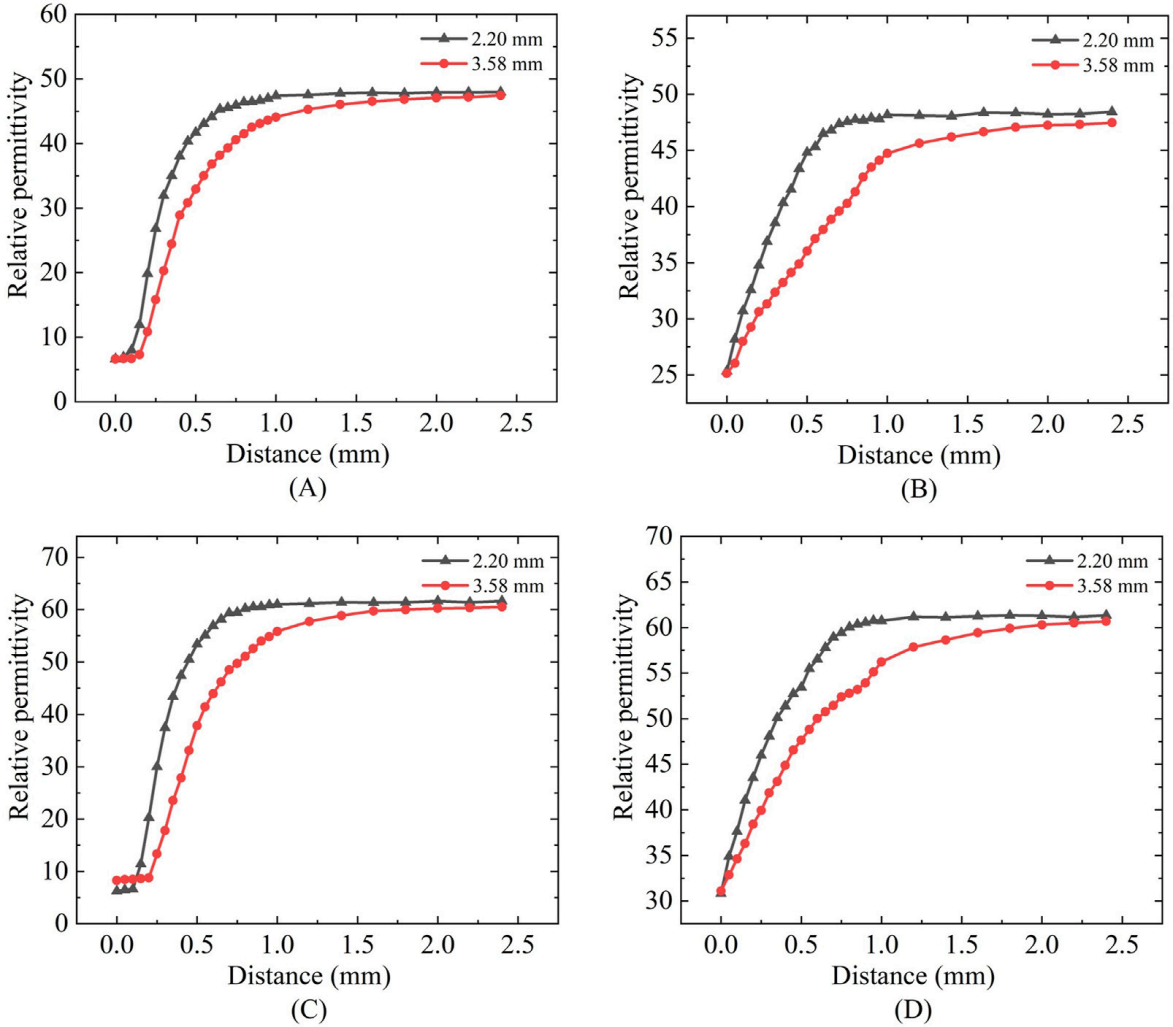
Mimic porcine tissue and mimic breast tissue measurements: **(A,C)** Sensing depth measurements; **(B,D)** Sensing radius measurements.

Based on the measurement results shown in Figures 12A,B it can be calculated that for the simulated porcine tissue, the effective sensing depth and radius of the 2.20 mm probe are 0.55 mm and 0.45 mm, respectively, whereas those of the 3.58 mm probe are 0.85 mm and 0.87 mm, respectively.

From Figures 12C,D it can be observed that for the simulated breast cancer tissue measurements, the effective sensing depth and radius of the 2.20 mm probe are 0.58 mm and 0.55 mm, respectively, while those of the 3.58 mm probe are 0.93 mm and 0.93 mm, respectively. Table 7 shows that there is a general increase in depth of sensing and radius of sensing as the probe size increases from 2.20 mm to 3.58 mm.

**TABLE 7:** Effective sensing volume of the two sizes of probes measured in real and simulated organizations.

Probe size	2.20 (mm)				3.58 (mm)			
Sensing volume	Depth (mm)	Standard deviation	Radius (mm)	Standard deviation	Depth (mm)	Standard deviation	Radius (mm)	Standard deviation
Porcine tissue	N/A	N/A	0.54	0.05	N/A	N/A	0.72	0.04
Breast cancer tissue	N/A	N/A	0.60	0.07	N/A	N/A	0.76	0.05
Simulated porcine tissue	0.55	0.03	0.45	0.03	0.85	0.03	0.87	0.02
Simulated breast cancer tissue	0.58	0.02	0.55	0.03	0.93	0.02	0.93	0.02

**FIGURE 13:**
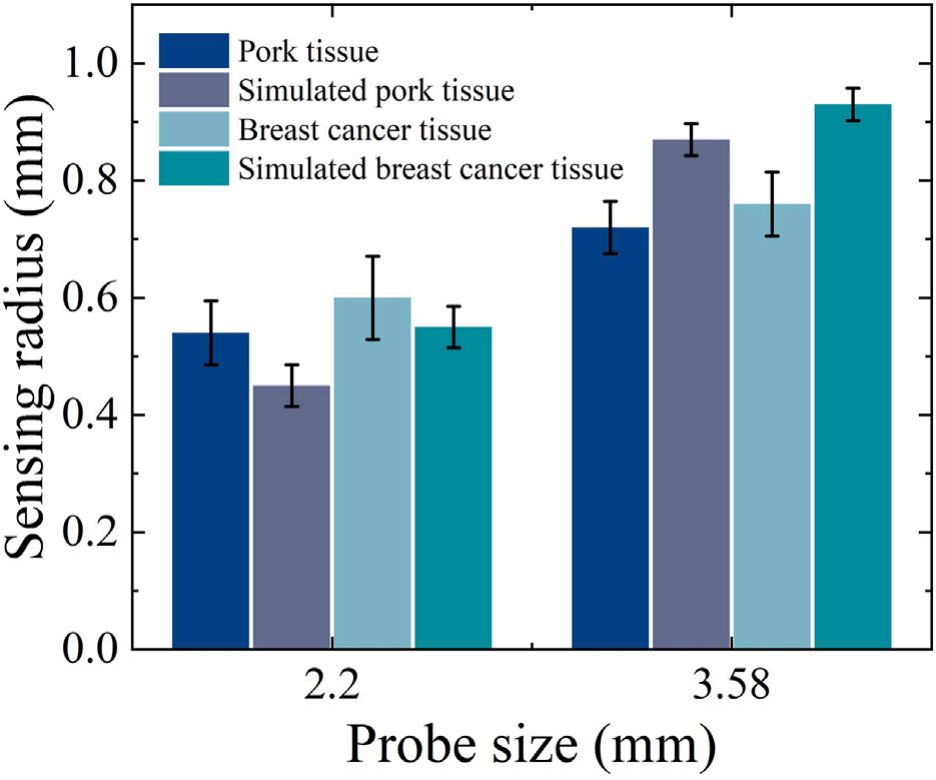
Effective sensing radius for real tissues and simulated tissues.

In Figure 13 it can be observed that the sensing radius obtained from measurements of biological tissues are largely consistent with those obtained from measurements of tissue-mimicking liquids. For the 2.20 mm probe, the error in the effective sensing radius between real tissue and tissue-mimicking liquid measurements is smaller than that for the 3.58 mm probe, indicating that the smaller probe offers higher measurement accuracy.

Regarding permittivity measurement consistency: Figures 6, 12 demonstrates that different probe sizes yield comparable permittivity values when measuring either homogeneous phantoms or real tissue blocks at identical frequencies. This aligns with theoretical expectations: when measuring homogeneous media or at sufficient distances (where double-layer effects diminish), probe size should minimally affect permittivity measurements. The convergence of measurement curves with increasing distance in Figures 6, 12 empirically confirms this theoretical framework.

## 5 Discussion

Measurement of the dielectric properties of biological tissues based on coaxial probe measurement systems has been a hot issue in the research field. Defining the sensing radius of small aperture probe sizes, and thus using appropriate probe sizes, is important for accurate measurement of dielectric properties of multilayered tissues, such as breast tissue.

To address this, we developed an accurate and reproducible measurement system and protocol to quantify the effective sensing depth and radius of small-aperture probes. By leveraging materials with distinct dielectric properties (such as Teflon, ethanol, methanol, deionized water, and tissue-mimicking solutions), we constructed a bilayer model with dielectric property gradients to represent heterogeneous tissue structures. The system was further validated through measurements of porcine tissue and human breast tissue.

Although Liu et al. previously utilized a comparable methodology for sensing radius measurements, their approach demonstrated inherent limitations in both accuracy and stability. Our enhanced apparatus represents substantial progress through three key innovations. First, the implementation of micrometer-based measurement achieves a resolution of 0.01 mm, representing an order-of-magnitude improvement over traditional caliper precision. Second, structural optimization incorporating integrated slide rails and sample clamps effectively reduces positional errors associated with manual operation. Finally, the system’s enhanced repeatability guarantees reliable data acquisition for micro-region characterization, high-precision experimentation, and *in-situ* measurement applications. Together, these technological advances enable the establishment of standardized measurement protocols with superior scientific rigor and enhanced cross-study comparability.

In this study, the depth of sensing was chosen to be defined as the distance at which the probe starts to move when the measured value is 90% of the dielectric characteristic parameter of the reference target (Tissue 2). In this experiment we set the geometric center of Teflon cube as the starting point of the probe movement. The measured sensing depth ranged from 0.44 to 0.62 mm for the 2.20 mm diameter probe and from 0.75 to 0.98 mm for the 3.58 mm diameter probe. Furthermore, using a sensing radius measurement device, the corresponding sensing radius was determined to range from 0.36 to 0.63 mm for the 2.20 mm diameter probe and from 0.71 to 0.99 mm for the 3.58 mm diameter probe.

In a 2003 study by Hagl et al., the sensing depth was investigated based on a 10% error threshold for measuring the dielectric properties of ethanol, methanol, and water. Their results indicated that the required sample thickness ranged from 0.75 to 1.5 mm for the 2.20 mm diameter probe and from 1.25 to 3.0 mm for the 3.58 mm diameter probe (Hagl et al., 2003). In 2020, Liu et al. measured the sensing radius for deionized water and ethanol, reporting values in the range of 1.0–1.6 mm (Liu et al., 2020). Both studies reported slightly larger values compared to our findings, which can be attributed to the use of different materials (Tissue 1) as the measurement starting point, as well as differences in the measurement setups. In this study, we employed a micrometer-assisted sliding rail method for sensing volume measurement, which provides higher accuracy and stability compared to traditional vernier caliper methods.

**FIGURE 14:**
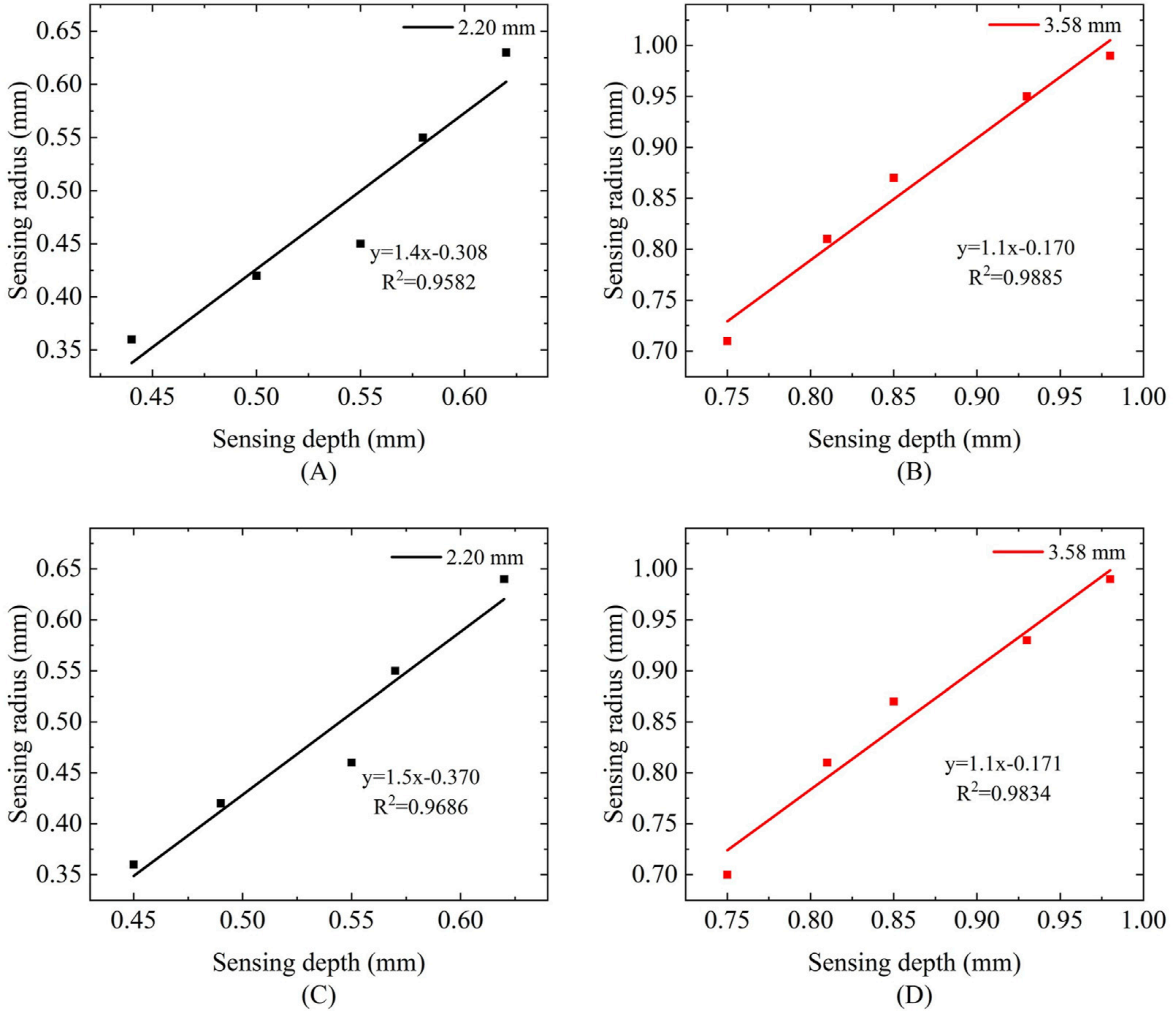
Relationship between sensing radius and sensing depth at different frequencies: **(A,B)** Frequency of 300 MHz; **(C,D)** Frequency of 500 MHz.

We observed that the variation in probe sensing depth is proportional to the variation in sensing radius. To further elucidate the relationship between the two, we performed linear fitting on the sensing volume of five measurement liquids. Figure 14 illustrates the relationship between sensing radius and sensing depth under different frequencies and probe sizes. In Figures 14A,C corresponding to a probe size of 2.20 mm at frequencies of 300 MHz and 500 MHz, the R^2^ values were 0.9582 and 0.9686, respectively, indicating a strong linear correlation between the fitted line and the data points. In Figures 14B,D corresponding to a probe size of 3.58 mm at the same frequencies of 300 MHz and 500 MHz, the R^2^ values were 0.9885 and 0.9834. Although the linear correlation is slightly lower compared to the 2.20 mm probe, it still demonstrates a high degree of linearity. Furthermore, the influence of different frequencies on the linear relationship is minimal.

In practical measurements, controlling the thickness of biological tissues poses significant challenges. Consequently, most current studies employ liquids that mimic the dielectric properties of tissues as substitutes to measure the effective sensing depth in heterogeneous tissues (Hagl et al., 2003; Meaney et al., 2014). While this approach eliminates the need for tissue segmentation, the use of liquid substitutes instead of real tissues may introduce biases in the measurement results. In this study, we enhanced measurement authenticity by directly measuring tissue samples cut to specific thicknesses. However, the issue of precisely controlling the thickness of the measured tissue remains unresolved. In the next phase, we plan to introduce more advanced measurement tools and techniques to achieve more accurate segmentation and measurement of the target Tissue 2.

To systematically evaluate tissue heterogeneity effects, we implemented a controlled bilayer model with incrementally increasing dielectric contrast: low contrast (Teflon, ε′ ≈ 2.1/Ethanol, ε′ ≈ 24, Δε′ ≈ 22), medium contrast (Teflon/Methanol, ε′ ≈ 33, Δε′ ≈ 31), and high contrast (Teflon/Deionized Water, ε′ ≈ 78, Δε′ ≈ 76). Experimental results demonstrate direct proportionality between dielectric contrast (Δε′) and sensing volume, where higher Δε′ values correlate with expanded sensing depth/radius (up to 38% increase in high-contrast scenarios), while low-contrast environments constrict the effective measurement zone.

These findings directly translate to clinical tissue structures, where dielectric properties at 300 MHz are well-established: fatty tissue (ε′ ≈ 5 (Gabriel et al., 1996)), glandular tissue (ε′ = 30–50 (Gabriel et al., 1996))and stromal tumor (ε′ ≈ 30 (Lazebnik et al., 2007)). When measuring interfaces like fat/stroma (Δε′ ≈ 25), sensing volume contracts significantly compared to fat/glandular boundaries (Δε′ ≈ 40), necessitating larger-aperture probes (≥3.5 mm) for low-contrast scenarios to mitigate positional errors, while smaller probes (≤2.2 mm) suffice for high-contrast regions.

As previously noted, the Figure 11 demonstrates close alignment in dielectric properties between phantoms and biological tissues across 300–1,500 MHz, validating our tissue-mimicking strategy. Subsequently, the consolidated Figures 12, 13 compare sensing performance in both phantom and biological systems, revealing that large-aperture probes exhibit slower response dynamics to distance variations than their small-aperture counterparts - consistent with their extended sensing range. Both phantoms offer optical transparency and mechanical stability for real-time monitoring while simplifying simulation parameterization due to their isotropic and homogeneous nature. However, their current homogeneous designs do not replicate microscopic heterogeneities present in biological tissues (e.g., muscle fiber structures or tumor microcalcifications). Future optimizations could incorporate 0.5% agarose to mimic cellular frameworks or disperse CaCO_3_ particles to simulate calcification sites - modifications shown to affect dielectric properties by <5% (Porter and O'Halloran, 2017), thereby enhancing structural fidelity while maintaining dielectric accuracy.

Our experiments also have many shortcomings; the initial position of the probe is difficult to control precisely, and because the experimental conditions are not carried out in an ideal state, there is usually a certain air gap between the probe and the measurement medium. This air gap can interfere with the measurement results and lead to inconsistencies in the measured initial values. In order to reduce the influence of this factor on the experimental results, it is necessary to optimize the experimental setup, improve the fixation of the probe or use compensation algorithms to reduce the initial value error as much as possible, so as to improve the reliability and repeatability of the experimental results.

Moreover, the two-layer hierarchical structure used in the experiment can provide a certain approximate model for the study, but it cannot fully reflect the complex heterogeneity characteristics and diverse structural differences in actual biological tissues. This also suggests that future studies need to adopt more complex models and experimental designs to more realistically simulate and measure the dielectric properties of heterogeneous biological tissues.

Probe diameter selection is clinically driven: evaluation of early-stage tumors necessitates shallow measurements (∼0.5 mm depth) to detect epidermal/dermal water content variations, whereas deeper adipose layer assessment (particularly in obese patients) requires greater penetration depths (∼5.0 mm). These depth parameters align with commercial TDC systems (Delfin Technologies), which offer 10–55 mm diameter probes achieving corresponding effective measurement depths of 0.5–5.0 mm at 300 MHz operating frequency (Toro et al., 2024). Future investigations will consequently utilize larger probes (≥5 mm diameter) for comprehensive deep-tissue characterization.

Although this study has established systematic trends in probe-size effects on sensing volume, several key variability factors warrant consideration: tissue heterogeneity arising from microstructural non-uniformities in biological tissues (particularly tumors) may cause local measurement fluctuations; probe-tissue interface variations in contact pressure and angular alignment during manual operation introduce positioning uncertainties; sample status factors including preservation conditions, temperature, and hydration levels affect *ex vivo* tissue dielectric properties; and modeling uncertainty stems from intrinsic fitting errors during S-parameter inversion. To address these limitations, future research will implement three critical improvements: increased sample sizes, triplicate measurements, and stricter experimental controls (e.g., fixed-contact-force mechanisms and optimized tissue preparation protocols). These substantive methodological enhancements will both clarify the scope of our current conclusions (based on observed systematic trends) and establish robust pathways for statistical validation in subsequent investigations.

## 6 Conclusion

This study compares the sensing depth and sensing radius of two different small aperture probes used for breast cancer detection. The measurement model of the sensing volume of the open-end coaxial probe method was developed by using the dielectric property hierarchical model combined with micrometer-assisted sliding rail device. The experimental results were verified by measuring porcine tissue and breast cancer tissue. The results show that the sensing depth range of the probe increases significantly with the increase of the coaxial probe size, while the probe sensing radius is also proportional to the probe aperture. A smaller probe aperture may help to reduce the effect of tissue heterogeneity on the measurement, and the effective sensing volume of the probe does not change significantly with frequency.

## Data Availability

The raw data supporting the conclusions of this article will be made available by the authors, without undue reservation.
